# Structural Phenomena Introduced by Rotary Swaging: A Review

**DOI:** 10.3390/ma17020466

**Published:** 2024-01-18

**Authors:** Lenka Kunčická

**Affiliations:** Faculty of Mechanical Engineering, Brno University of Technology, Technická 2896-2, 616 69 Brno, Czech Republic; kuncicka@fme.vutbr.cz; Tel.: +420-541-142-507

**Keywords:** rotary swaging, intensive plastic deformation, microstructure, grain size

## Abstract

Rotary swaging is an industrially applicable intensive plastic deformation method. Due to its versatility, it is popular, especially in the automotive industry. Similar to the well-known methods of severe plastic deformation (SPD), rotary swaging imparts high shear strain into the swaged materials and thus introduces grain refinement down to a very fine, even ultra-fine, level. However, contrary to SPD methods, one of the primary characteristics of which is that they retain the shapes and dimensions of the processed sample, rotary swaging enables the imparting of required shapes and dimensions of workpieces (besides introducing structure refinement and the consequent enhancement of properties and performance). Therefore, under optimized conditions, swaging can be used to process workpieces of virtually any metallic material with theoretically any required dimensions. The main aim of this review is to present the principle of the rotary swaging method and its undeniable advantages. The focus is primarily on assessing its pros and cons by evaluating the imparted microstructures.

## 1. Introduction

### 1.1. Grain Refinement Mechanisms

Rapid developments in engineering, together with the demand for innovative materials featuring specific properties for challenging applications, from transportation [1,2], through power engineering [3,4,5], to biomedicine [6,7], have resulted in the emergence of several paths leading to the fabrication of various metallic materials with exceptional performances and increased longevity. The mechanical properties—ultimate tensile strength (UTS), in particular—of metallic materials are highly influenced by the microstructures, i.e., grain size and morphology [8,9]. In accordance with the Hall–Petch law, the strength of a polycrystalline material generally increases with decreasing grain size [10]. However, the structure refining processes differ according to the intrinsic material characteristics, especially the stacking fault [11,12].

For metals featuring high stacking fault energy (e.g., aluminum), crystallographic defects, such as dislocations, form within the microstructure due to the effects of an external force, which is greater than the flow stress of the material. As the external force continues its action, the generating dislocations start to move through the material, preferably along the available slip systems. With increasing dislocation density, they start to accumulate in dislocation tangles and cells, which further form dislocation walls. Together with this, microbands start to occur in locations in which more slip systems (usually two independent sets) are activated [13]. Subsequently, lamellar subgrains, defined by low-angle grain boundaries (LAGBs), featuring high densities of dislocations are formed. Due to the effect of the continuing accumulation of dislocations at the subgrains’ boundaries, these boundaries increase their misorientations up to the level of high-angle grain boundaries (HAGBs), resulting in full new grains (lamellar and/or equiaxed) developing.

For metals featuring low stacking fault energy (e.g., copper), the grain refining mechanism starts with the generation of dislocations and their movement along the preferred slip systems, similar to metals featuring high stacking fault energy. However, within these metals, the acting external force also imparts the (substantial) generation of other defects, such as twins and stacking faults [14]. The generated dislocations then accumulate at twin boundaries (being strong barriers for dislocations movement), resulting in the originally coherent twin boundaries gradually transforming into semi-coherent ones. With the continuing accumulation of the imposed strain, the original twin boundaries gradually transform into regular HAGBs, and relatively long lamellar grains are formed. Together with this, the aforementioned phenomena continue to occur within the material as the progressive deformation still generates new dislocations, twins, and stacking faults. The continuous interaction of primary and secondary dislocations, twins, and stacking faults, together with the gradual occurrence of structure-forming phenomena, such as grain rotation [15], and grain boundary sliding [16], then finally introduce the formation of UFG or nanostructures within the metallic material. For some metals with low stacking fault energy, especially those featuring the FCC lattice, the structure-refining processes can be even more complicated, as twinning can be combined/interact with other structure-forming phenomena, including secondary de-twinning (e.g., [17]).

The key point of all the mentioned grains’ evolutions chains is, however, that dislocations tend to accumulate at defects, among which, especially, are grain (twin) boundaries [18]. Therefore, the higher the number of crystallographic defects, the greater the flow stress and thus strength of the metallic material. In other words, a higher fraction of grain boundaries represents a higher number (volume fraction) of obstacles for dislocation movement and thus the finer are the grains occurring within the material, and the higher is the (yield, ultimate) strength [19]. Nevertheless, this only applies to a certain limiting value of grain size (~10 nm), from which the volume fraction of the grain boundaries within the material gradually becomes incomparably higher than the volume fraction of the grain interiors. At this point, the grain boundary sliding (GBS) phenomenon starts to gain significance [20]. This increases the plasticity of the material and facilitates plastic deformation. Therefore, continuing to decrease the grain size below this critical level (if possible) also results in decreasing the strength of the material [21]. This phenomenon, occurring at very low (nano) grain sizes, is referred to as the inverse Hall–Petch relationship [22].

### 1.2. Methods of Grain Refinement

Polycrystalline metallic materials can be characterized into categories according to the grain size: coarse-grained (grain size > 10 µm), fine-grained (grain size 1–10 µm), ultra-fine grained (grain size 0.1–1 µm), and nano-sized (grains < 100 nm) [23]. Keeping in mind the Hall–Petch law, especially the ultra-fine grained (UFG) and nano-materials have been intensively researched (e.g., [24,25]). To achieve the final grain size in such small scales, numerous methods of severe plastic deformation (SPD)—for example, Equal Channel Angular Pressing (ECAP) [26,27,28,29,30]—various ECAP modifications [31,32,33,34,35,36,37,38,39]—High Pressure Torsion (HPT) [40,41], Friction Stir Processing (FSP) [42], or Accumulative Roll Bonding (ARB) [43,44]—and intensive plastic deformation, i.e., IPD, methods (e.g., asymmetric reduction rolling [45,46], (high speed ratio) differential speed rolling [47], and asymmetric extrusion [48], etc., have been introduced. In principle, the methods are based on imposing high, or extreme, shear strain into the processed (metallic) materials, the effect of which being that they impart (substantial) grain refinement via introducing lattice distortions and crystallographic defects leading to the development and accumulation of dislocations according to the above-described schemes (depending on the intrinsic properties of the metallic material).

Nevertheless, the ability to introduce a highly efficient grain refinement is not the only advantage of the IPD and SPD shear strain-based methods. Among their advantages are also that they introduce predominantly compressive stress states, can be optimized to modify/control the state of residual stress [49,50], enable the reduction/elimination of the presence of voids and residual porosity [51], and many more. Therefore, IPD and SPD methods are also highly advantageous for processing materials with challenging formability [52,53], multiphase materials [54,55,56], shape memory alloys [57,58], or various composites [59,60]. Moreover, the generally advantageous combination of compressive stress state and high shear strain is also favorable for the direct consolidation of powders [61,62].

## 2. Rotary Swaging Method

**Figure 1:**
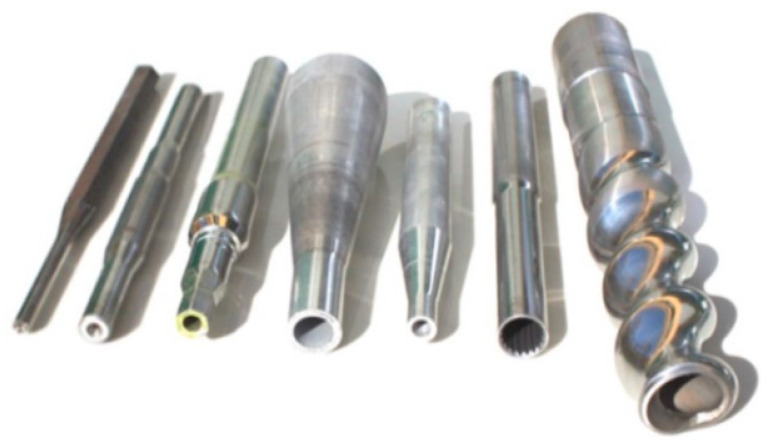
Examples of final components manufactured by RS [original by author].

Rotary swaging (RS) is an IPD method, which is industrially applicable (typically in the automotive industry today) [63,64]. Similar to the commonly known SPD methods, the basic principle of RS lies in the gradual application of high shear strain increments into the processed metallic material with the aim of achieving (severe) grain refinement. The mentioned predominantly compressive stress state during swaging contributes not only to grain fragmentation but also the homogenization of residual stress. However, certain differences between the SPD and RS methods can be identified; these are primarily in the (limits of) workpiece dimensions [65,66]. Contrary to SPD methods, RS imparts changes in the dimensions of the processed workpieces and thus also enables the achievement of (more or less complicated) components of various (challenging) geometries. Examples of components manufactured by RS can be seen in Figure 1. Moreover, the maximum volume (i.e., length) of the processed workpiece is theoretically unlimited. In other words, RS is so versatile that it can be used to process workpieces with virtually any required final dimensions from virtually any metallic material; the only limits of its applicability lie in the used dies and the power output of the engine [67]. It is suitable to manufacture components with cross-sections varying from circular to complex, all with finished surfaces of exceptional quality without any necessity for final grinding or polishing (see Figure 1).

**Figure 2:**
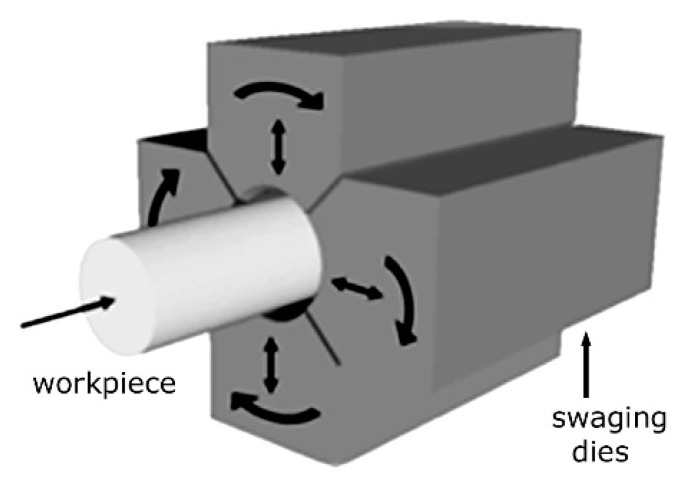
Schematic depiction of swaging dies assembly [original by author].

The strain increments are imparted by a set (typically four pieces) of rotating dies assembled in a swaging head in a circumferential manner around the workpiece, as schematically depicted in Figure 2. By switching on the engine of the machine, the swaging head with the dies starts to rotate around the inserted workpiece with a maximum speed of up to 2000 strokes per minute. Together with the rotational movement, the dies also periodically move towards and outwards the rotational axis of the swaging head (the workpiece in which is located), as depicted in Figure 2. This movement, which actually provides the deformation of the workpiece, is ensured by the effect of centrifugal force. Such configuration is suitable for the swaging of axisymmetrical components, typically with circular cross-sections. For the swaging of components with complex shapes and non-circular cross-sections, additional features can be present, if necessary, for the successful fabrication of a component with a specific design (auxiliary engines, CNC operation, hydraulic mandrels, etc.) [68].

**Figure 3:**
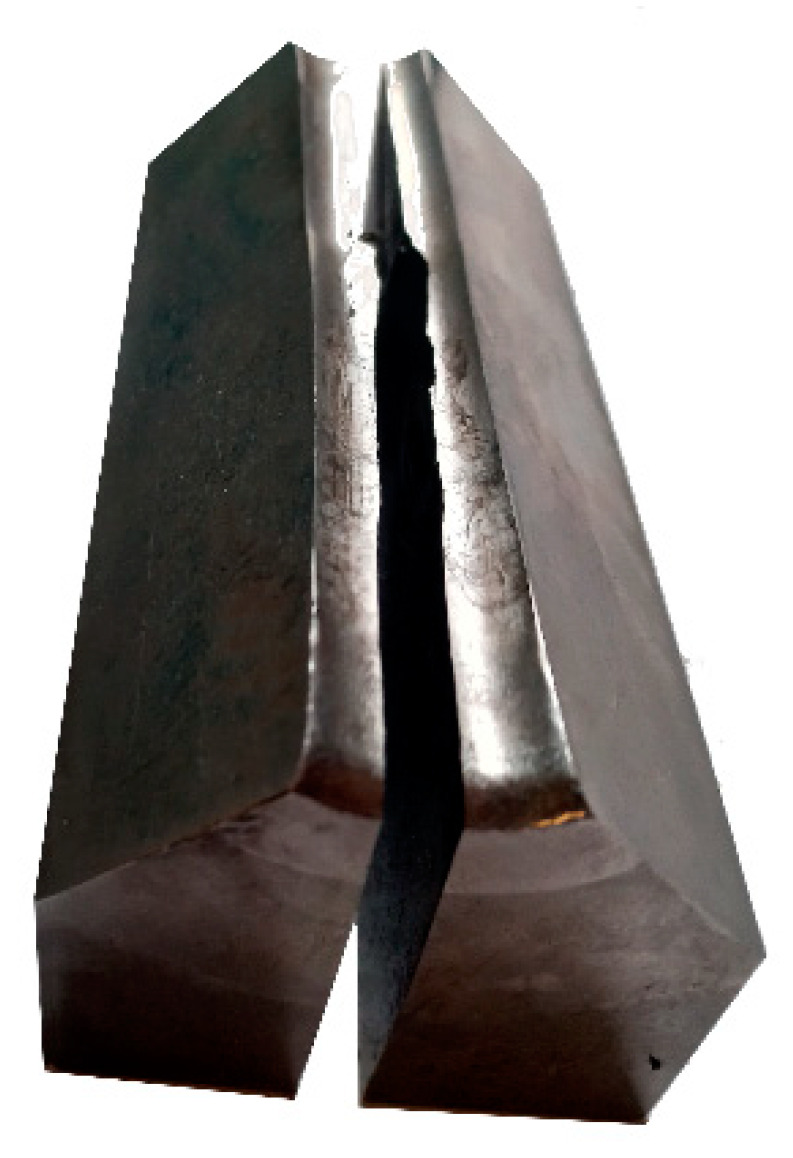
Damage to dies occurring due to unsuitably selected swaging conditions [original by author].

In order to successfully perform RS, the processing parameters, as well as the geometries (and number), of the used swaging dies need to be optimized while considering the processed material, intended swaging temperature, and desired total swaging reduction (final shape of the component). Among the parameters worth considering are primarily the rotational speed, feed rate, friction, and, last but not least, processing temperature. If the selected swaging parameters are not suitable for the required processing technology or are selected inappropriately with regards to the processed metallic material, numerous defects to the workpiece (cracks, bulging, spiral ridges, flashes, etc.), but also to the machine (cracking of dies, wear of rollers, etc.), can occur; see Figure 3 depicting an example of possible defects that can occur to the dies. Moreover, the unsuitable selection of processing conditions, such as swaging temperature or reduction steps, can lead to the destruction of workpieces, especially those prepared from brittle metallic alloys or from original powders canned for direct consolidation [69].

Having selected the processing conditions with regard to the material of the processed workpiece and required shape changes, another viewpoint has to be considered—the (total as well as incremental) imposed strain—as it does not only directly affect the microstructures of the swaged components (and thus final properties) but also the deformation behavior of the material during swaging. Therefore, the processing conditions, and especially the swaging temperature, must also be considered in relation to the imposed strain. The swaging ratio, i.e., the total shear strain imposed during swaging, can be calculated via (Equation (1)),

(1) $$\varphi =\ln\left(\frac{S_{0}}{S_{n}}\right)$$

where S_0_ and S_n_ are cross-sectional areas of the workpiece at the input and output of the swaging dies, respectively.

**Figure 4:**
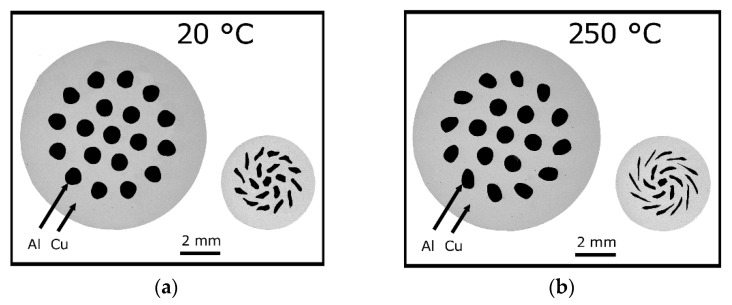
Cross-sectional cuts through rotary-swaged Al/Cu-clad composites showing their deformation behaviors: 20 °C, *φ* = 2.2 (**left**) and *φ* = 3.6 (**right**) (**a**); 250 °C, *φ* = 2.2 (**left**) and *φ* = 3.6 (**right**) (**b**) [original by author].

Due to the nature of the swaging process, the imposed strain is homogeneously distributed across the cross-sections of the swaged workpieces, especially for lower total swaging ratios. In other words, as the swaging dies affect the workpiece from its periphery, the highest imposed strain is typically in the (sub)peripheral region of the workpiece and its intensity decreases across its cross-section towards its axis [70]. This inhomogeneity gradually decreases with increasing swaging ratios, and can also be diminished by increasing the swaging temperature. Figure 4a,b shows an explicit example. Figure 4a depicts a cross-sectional cut through of an Al/Cu-clad composite swaged with a total swaging ratio of 2.2 (left), as well as a cross-sectional cut through of an identical Al/Cu-clad composite swaged with a swaging ratio of 3.6 (right, both swaged at room temperature). Note that increasing the swaging ratio contributed to the penetration of the imposed strain towards the axial region of the composite workpiece, i.e., the Al wires inserted within the Cu matrix were more affected by the swaging when the swaging ratio increased. However, see also in Figure 4b, the cross-sectional cuts through an identical type of the Al/Cu-clad composite swaged at the elevated temperature of 250 °C with the swaging ratios of 2.2 (left) and 3.6 (right). Evidently, the increased swaging temperature decreased the flow stress of the individual components of the processed composite and supported the penetration of the imposed strain towards the axial region of the workpiece even at relatively lower swaging ratios (note that the Al wires of the composite were already visibly deformed at the swaging ratio of 2.2 when the swaging temperature was elevated to 250 °C, Figure 4b) [71,72].

Keeping in mind the possible variations provided by the possibilities of alterating the imposed strain (and other processing conditions), and their direct relation to the development of microstructure and properties [73,74], the swaging process can be tailored to introduce various microstructures according to the desired requirements. It is even suitable to create gradient structures [75,76].

## 3. Microstructure Development

As indicated, RS is highly favorable for the preparation of UFG microstructures. However, the deformation behaviors of processed metallic materials differ, primarily according to the (available) activated slip systems, i.e., crystallographic lattice.

### 3.1. FCC Lattice

#### 3.1.1. Single-Phase Metallic Materials

FCC metals exhibit a high number of available slip systems and thus generally feature favorable plasticity. Among the commonly investigated FCC metals are, e.g., copper or aluminum and their alloys.

**Figure 5:**
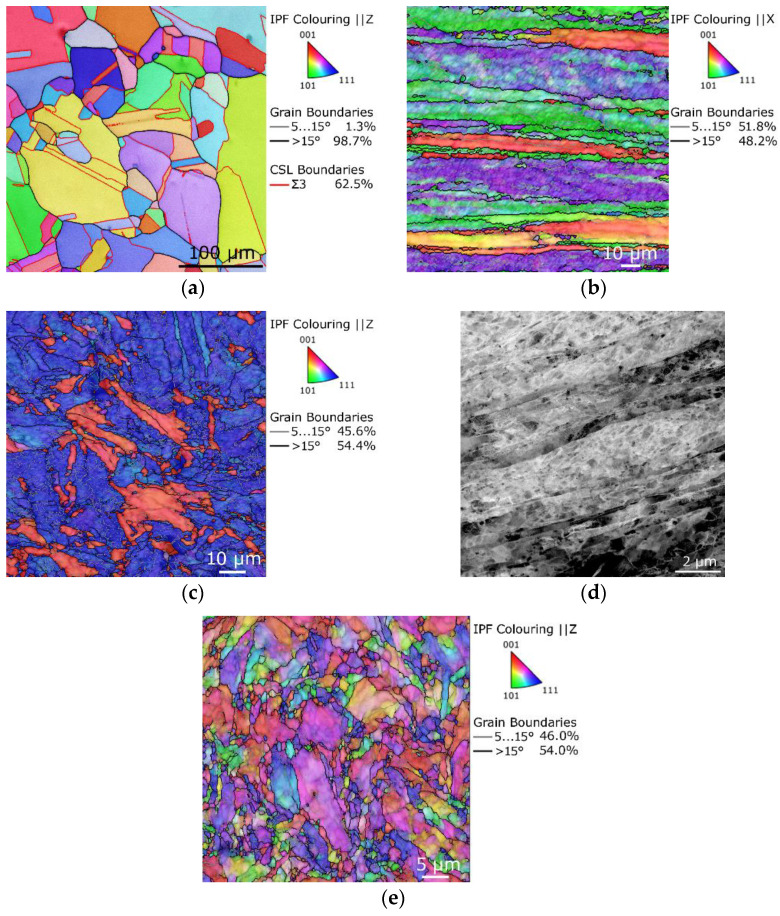
OIM of conventional annealed CG Cu (**a**), OIM of Cu swaged at room temperature, *φ* = 2.8, longitudinal cut (**b**); OIM of Cu swaged at room temperature, *φ* = 2.8, cross-sectional cut (**c**); TEM dark field image of substructure from the longitudinal cut in 5 (**b**,**d**); OIM of Cu swaged at 250 °C, *φ* = 2.8, cross-sectional cut (**e**) [original by author].

Taking Cu as the first example, Cu in an annealed state typically features coarse grains (CG) with random orientations and the presence of numerous twins; Figure 5a depicts an orientation image map (OIM) of a typical CG Cu structure with an average grain size of 37.4 µm (determined as max. Feret diameter). When subjected to high shear strain via RS, lattice defects and twins are generated according to the scheme presented above for low SFE metals and the original coarse grains start to gradually elongate in the direction of the dominant plastic flow vector [77,78]. Figure 5b depicts the OIM of a longitudinal cut, and Figure 5c shows the OIM of a cross-sectional cut through a Cu bar swaged at room temperature with a ratio of 2.8; the average grain size at the cross-section of the swaged Cu was ~ 3.8 µm. RS thus contributed to the significant refinement of grains within the Cu (grains’ cross-sections reduced approx. ten times for the given swaging ratio). 

After a sufficiently high accumulation of strain, restoration processes are activated. As Cu features low SFE, the annihilation of dislocations and subsequent restoration typically occurs before dynamic recrystallization takes place and thus the deformed microstructure typically features dislocation substructures with high densities of dislocations and substantial volume fractions of dislocation tangles and cells (see the example in Figure 5d, transmission electron microscopy (TEM) image taken from the longitudinally cut sample of the OIM image that was depicted in Figure 5b). Similar microstructure development was observed by, e.g., Kopeček et al. [79], who achieved mutually enhanced mechanical and electric properties by imparting severely elongated grains, the majority of which having cross-sections smaller than 5 µm at the maximum swaging ratio of 3.2, with developed dislocation substructures and <111> and <100> || swaging direction fiber textures of high intensities via room temperature RS. 

The development of dynamic recrystallization within swaged Cu can be supported by increasing the processing temperature; compare the OIM image in Figure 5c with the OIM image in Figure 5e showing a cross-sectional cut through a Cu bar swaged at the elevated temperature of 250 °C with the identical swaging ratio of 2.8. The microstructure in Figure 5e featured bimodal grain distribution, and coarse grains could still be observed but together with a high fraction of newly emerging recrystallized ones. Dynamic recrystallization thus occurred at a lower level of the accumulated shear strain, i.e., at a lower swaging ratio, for the elevated processing temperature as the texture intensity diminished significantly (see the more or less random coloring of the individual grains in the Figure).

In order to further optimize the microstructures, (commercially) pure metals can be alloyed to introduce additional secondary phases and/or formations of solid solutions, but the RS processing itself can also be combined with thermomechanical or annealing treatments. For example, Huang et al. [80] and Martynenko et al. [81] used RS in combination with ageing to achieve homogeneous distributions of Cu_5_Zr precipitates, increase the UTS up to ~600 MPa, and enhance the overall performance of a CuCrZr alloy. Martynenko et al. [82] performed a study in which they subjected a CuHf alloy to various swaging ratios and subsequent ageing treatments to optimize the ratio of its mechanical and electric properties; by applying the *φ* of 2.77 and ageing at 475 °C for 2 h, they achieved a microstructure with nano-sized subgrains (~180 nm) featuring the UTS of ~460 MPa and electric conductivity of almost 91% IACS (International Annealed Copper Standard). Ouyang et al. [83] applied cold RS and subsequent ageing to control the precipitation of nano-sized ordered phases within a CuNiSnNb alloy and consequently achieve a UTS of 1230 MPa (however, at the expense of ductility). Zhao et al. [84] combined RS with annealing at various time dwells and temperatures (up to 120 min and 400 °C) to optimize the distribution of UF lamellar α-Cu solid solution and Sn-rich phases to reduce the risk of development of microcracks, and Cao et al. [85] used cold RS as a tool to promote deformation-induced melt activation inducing the formation of spherical fine grains within a C5191 Cu alloy.

**Figure 6:**
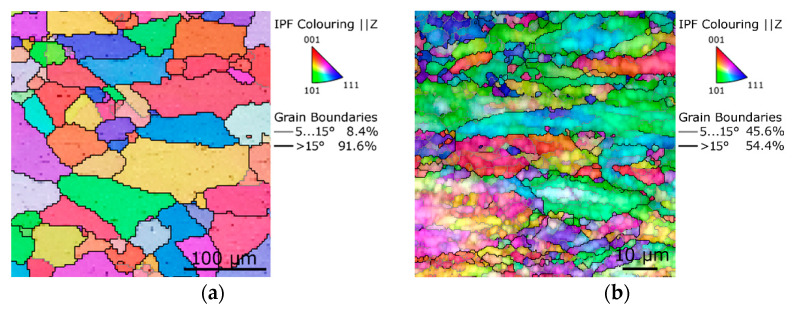
OIM of conventional annealed CG Al (**a**), OIM of Al swaged at room temperature, *φ* = 2.8, longitudinal cut (**b**) [original by author].

As regards the texture development within swaged Cu (alloys), the RS process tends to impart (more or less intense, according to the processing conditions) <100> and <111> fiber textures, as confirmed by, e.g., the OIM images in Figure 5c,e. Similar texture development during RS can also be observed for another FCC metal, i.e., aluminum. However, as Al features different intrinsic properties (especially the SFE), the microstructure development during RS is thus slightly different than for the Cu, primarily as regards the occurrence of dynamic recrystallization. Having a high SFE, original annealed Al also features a CG structure, but the occurrence of twins is negligible/none (see Figure 6a depicting an OIM image of a CG Al with the original average grain size of 69.5 µm). When subjected to high shear strain, the grains within Al tend to recrystallize more easily than within Cu, and thus Al subjected to comparable swaging ratios typically features lower average grain size than Cu, see Figure 6b showing an OIM image of a microstructure taken from a longitudinal cut through an Al rod subjected to the swaging ratio of 2.8 at room temperature (i.e., identical conditions as applied for the Cu, the microstructure of which was depicted in Figure 5b). The microstructure is evidently highly refined compared with that of Cu (Figure 5b) and features a high fraction of newly recrystallized equiaxed grains and well-developed dislocations substructure, which is evident from the shadings of colors in the individual larger grains in the OIM. For Al subjected to RS, the grains typically refine to a certain level and then exhibit a more or less steady state. Similar conclusions were also drawn by Abdulstar et al. [86], or Yang et al. [87], who reported commercially pure Al to exhibit a relatively uniform microstructure after being subjected to a swaging ratio of about 2.

Similar to Cu, Al can also be alloyed and further thermomechanically processed to achieve UFG microstructures with precipitated phases in order to enhance the mechanical properties and lifetime of the metallic material. Nokhrin et al. [88] performed a thorough study in which they treated AlZr alloys with additions of Si, Nb, and rare earth elements via the SPD method of ECAP followed by RS and annealing to introduce the formation of the UFG microstructure with the average grain size of ~2 µm and nano-sized Al_3_(Zr,Hf,Er) precipitates, therein achieving a Vickers microhardness that exceeded 480 HV. Similarly, Bochvar et al. [89] used combinations of cold ECAP, cold RS, and ageing to prepare AlMgSi alloys with additions of Sc, Hf, and Zr featuring optimized nano-sized precipitates of β and β’ secondary phases and a UTS reaching to 400 MPa. Jin et al. [90] applied solution treatment and ageing in combination with RS to an AlMgSiCu alloy. By increasing the amount of imposed shear strain, they did not only impart the formation of the UFG microstructure, but via the continuous generation and accumulation of dislocations and their interaction with precipitates, they achieved nano-sized precipitation leading to an increase in the UTS to more than 410 MPa. Lourenço et al. [91] used directional solidification together with 91% swaging reduction and recrystallization annealing to optimize corrosion behavior, i.e., increase the open circuit potential, of an AlCu alloy. Lin et al. [92] used RS combined with solid solution treatment to enhance the work hardening ability of an 2024 Al alloy, and the work hardening exponent for the alloy increased from 0.193 (as-fabricated) to 0.311 (solution treated at 510 °C for 2 h and rotary swaged).

#### 3.1.2. FCC Composites

Given the abovementioned advantages, RS is also highly favorable for the preparation of composites of various types. The mutual effect of high shear strain and compressive stress state supports not only diffusion bonding but also mechanical bonding of different metals/phases. Therefore, RS can also advantageously be used to fabricate laminates and clad composites. Among the clad composites consisting of metals featuring the FCC lattice are those combining Al and Cu. As wires manufactured from this combination of metals are very promising for prospective usage as electric conductors with decreased weight and increased longevity, they have been widely researched and fabricated in various types, from relatively simple designs (Cu tube with Al core [93] and initial tubes inserted within–two layers [94] and three layers [95]), through sheets with inserted filaments (both Al sheet + Cu filaments [96] and Cu sheet + Al filaments [97]), to complex designs (Al sheet with peripheral Cu lamellas and Cu core [98,99]). 

**Figure 7:**
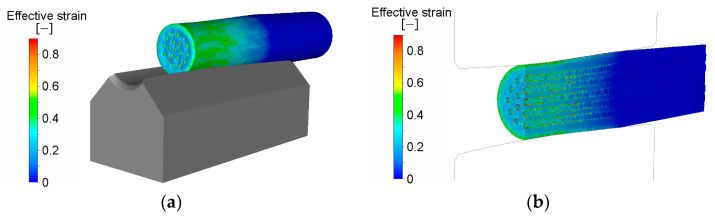
Distribution of effective imposed strain (**a**); effective imposed strain along axial longitudinal cut through composite (**b**) (by author, previously published in [100]).

As for such composites, the microstructure development within the individual metals is comparable to single-phase metals. However, certain differences can be observed due to differences in the plastic flows of the individual components—especially for laminates, as the distribution of the metallic components across their cross-sections is typically not uniform (see the distribution of the imposed strain during rotary swaging of a clad composite consisting of Al sheath and Cu wires, predicted via the finite element method, in Figure 7a,b [100]).

**Figure 8:**
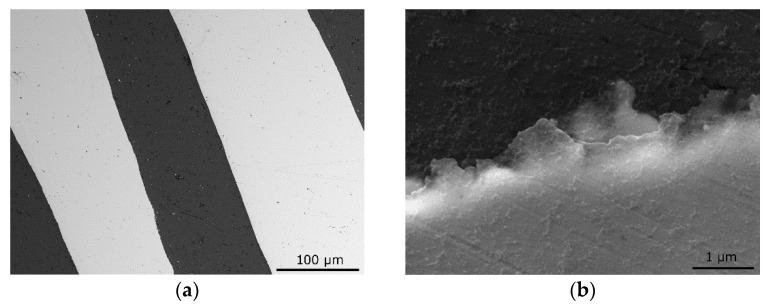
SEM-BSE image of interfaces within Al + Cu laminate swaged at: room temperature (**a**); 300 °C (**b**) [original by author].

For example, for a laminated rod consisting of Al-Cu-Al layers subjected to room temperature RS with a swaging degree of 2.2, different structure-forming phenomena within the Al layers were observed, according to its particular location [95]. Within the inner Al core, narrow bands of refined grains of widths ranging between 2 μm and 25 μm (sizes of individual grains) were observed—these bands further featured the presence of subgrains with sizes from 0.2 μm to 1.0 μm. On the other hand, the grains detected within the outer Al layer were larger but with a more substantial development of subgrains. Compared with the inner Al layer, the outer one was subjected to a greater amount of imposed shear strain, and thus the chain of strain hardening–softening microstructure phenomena developed to a greater extent therein. Similar variations between external and internal layers of the same metals were also confirmed for different types of swaged laminates (e.g., for Cu lamellas and Cu core of a laminate featuring an Al sheath [99]). Moreover, by varying the processing conditions, the optimization of the properties of mutual Al–Cu interfaces can be achieved [94,100,101], see also Figure 4a,b. Thorough research of the interfaces of Al + Cu-clad composites fabricated via rotary swaging revealed that swaging at room temperature results in the perfect bonding of the individual component layers with no development of brittle intermetallic phases. Compare Figure 8a, showing a SEM backscatter electron image of the interfaces within an Al + Cu laminate swaged at room temperature with the swaging ratio of 2.2 to Figure 8b showing a detailed SEM backscatter electron image depicting the development of mixed phases at the interfaces within an Al + Cu laminate swaged with a swaging ratio of 2.2 at a temperature of 300 °C. The intermetallic phases typically observed at such interfaces comprise (according to increasing Cu content) AlCu, Al_2_Cu, Al_3_Cu_4_, Al_2_Cu_3_, and Al_4_Cu_9_ compounds.

Cu has also advantageously been combined with other metals to prepare various composites, for example Tian et al. [102] combined Cu with Mg to fabricate a laminated wire via cold RS; by applying a reduction of up to 71%, they achieved wires with the UTS reaching 290 MPa and an electric conductivity of up to 81.1% IACS. Yu et al. [103] used RS at 950 °C to manufacture a W-Cu composite from original powders, the deformation processing not only provided favorable strength but also ensured the density exceeding 99%, whereas Kocich et al. [62] used RS at both room and elevated (400 °C and 600 °C) temperatures to fabricate Cu composites reinforced with Al_2_O_3_ particles featuring UFG microstructures (average grain size refined down to 1.2 µm^2^ for the composite swaged at room temperature). An UFG composite combining Al_2_O_3_ particles and an Al matrix was prepared at room temperature RS by Kunčická et al. [60]. Lu et al. [104] prepared a UFG AlN_p_/Al composite with a hierarchical microstructure using cold RS, and its UTS exceeded 410 MPa primarily due to a homogeneous distribution of nano-sized AlN precipitates. Similar conclusions for this type of material were drawn by Nie et al. [105].

### 3.2. BCC Lattice

#### 3.2.1. Single-Phase Metallic Materials

**Figure 9:**
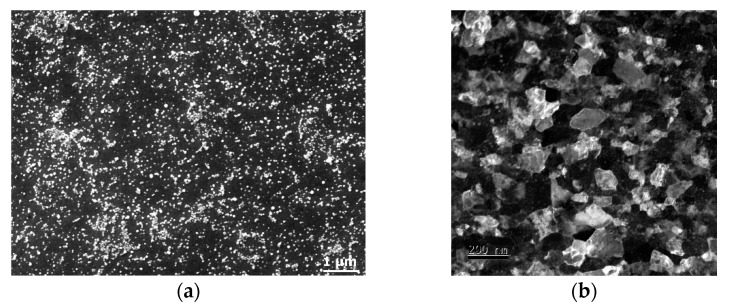
SEM-SE image of microstructure of ferritic steel strengthened with nano-sized Y_2_O_3_ particles (**a**); TEM image of microstructure of the Y_2_O_3_-strengthened ferritic steel (**b**) [original by author].

Contrariwise to FCC metals, metals featuring the BCC lattice are not so common with regards to experiments involving RS. Barkov et al. [106] applied RS to study the deformation behavior of W and Mo bars, while Wang et al. [107] prepared W cathodes with nano-sized Th from original powders via hot RS. Reith et al. [108] alloyed W with Re and La_2_O_3_ to enhance its hardenability and suppress recrystallization and further applied RS with a reduction exceeding 90% to achieve a UFG microstructure with exceptional creep resistance, and Liu et al. [109] combined the sol–gel method with high-temperature RS to fabricate W strengthened with nano-sized Y_2_O_3_ particles featuring microstructures stable up to 2 300 °C. Creep-resistant ferritic steel strengthened with nano-sized Y_2_O_3_ particles was directly consolidated via hot RS from a mechanically alloyed mixture of powders; the achieved Vickers microhardness of the bar subjected to a swaging ratio of 1.4 exceeded 700 HV1 [61]. The microstructure of such consolidated steel is depicted in Figure 9a (SEM-SE image) and Figure 9b (detailed TEM dark field image).

**Figure 10:**
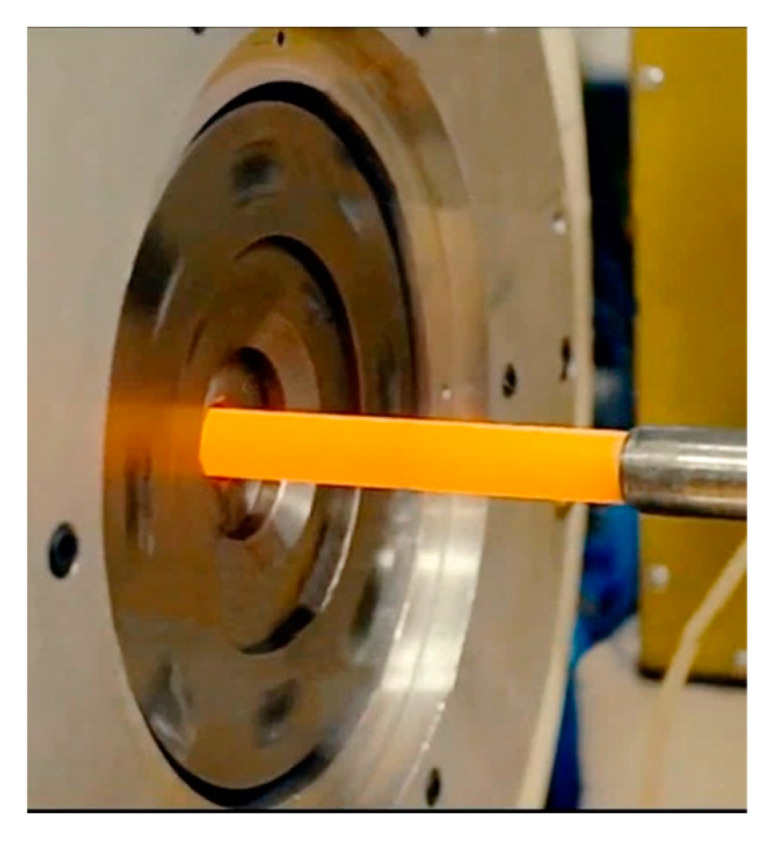
Rotary swaging of NiTi workpiece at 800 °C [original by author].

Xing et al. [110] used room temperature RS to deform a β-type TiNbTaZrO alloy and achieved a heavily hardened microstructure featuring a substantial volume of twins and the <110> fiber texture. Naydenkin et al. [111] applied RS in combination with subsequent ageing to achieve a supersaturated solid solution of β-Ti with nano-sized lamellar precipitates. Similarly, Thirathipviwat et al. [112] subjected a TiNbHfTaZr high entropy alloy (HEA) to a 90% reduction by RS at room temperature to acquire an ultimate strength exceeding 1000 MPa by accumulating a very high dislocations density (~10^15^ m^−2^). Silva et al. [113] applied RS with subsequent annealing at 900 °C and quenching to a bioapplicable β TiNbZr alloy to achieve the homogeneous distribution of the α’ martensitic phase, by which they achieved a decrease in the Young modulus down to 75 GPa. The effects of swaging on modifications in the Young modulus of bioapplicable β Ti-based alloys were also studied, e.g., by Hanada et al. [114], Jung et al. [115] (both TiNbSn alloys), or Baptista et al. [116] (TiNbZr alloy). Among the Ti-based BCC alloys, the performance of which can advantageously be influenced by RS processing, are also NiTi shape memory alloys [117]; see a photo of a NiTi workpiece being swaged at 800 °C in Figure 10. Wang et al. [118,119] performed research in which they studied the effects of RS thermomechanical processing on lattice parameters and the shape memory effect of a NiTi alloy; they described in detail the evolution of lattice rotation, strain field, and phase transformations. However, many of the metallic materials featuring (pre-dominantly) the BCC lattice are multi-phase materials or composites.

#### 3.2.2. BCC Composites and Multi-Phase Materials

As regards BCC multi-phase metallic materials, tungsten heavy alloys (THA) are among those subject to research involving RS the most frequently. THAs are typically prepared from mixtures of powders consisting of about 90 wt.% of W and other elements, such as Ni, Co, or Fe [120,121]. The powders are usually (cold) pressed and pre-sintered before further (deformation) processing. Given its incremental character and prevailing stress state, RS is highly favorable for the processing of such powder-based materials as it allows for the mutual elimination of residual porosity and the enhancement of mechanical properties. The alloys typically consist of W agglomerates and a matrix formed by additional (binding) elements. As regards THAs, RS has been performed for several types of chemical compositions and under various conditions. 

The microstructure development is generally comparable for all types of chemical compositions. Typically, the matrix is the more ductile phase, which deforms with greater ease. Strunz et al. [122] documented that the grain size of the NiCo2W matrix phase within a WNiCo pseudoalloy subjected to RS refined down to the UFG scale (~1.3 µm for cold RS, and ~1.0 µm for RS at 900 °C). The grains within this phase thus start to deform already at the beginning of deformation and, therefore, typically easily undergo recrystallization, especially when combined with further heat treatments. This statement was confirmed, for example, by Panchal et al. [123], who applied RS in combinations with annealing and quenching to THAs and reported the presence of recrystallized grains with a very weak texture within the matrix. Nevertheless, the accumulation of dislocations within the matrix phase supporting strengthening can be achieved by room temperature swaging with relatively low swaging ratios. Durlu et al. [124] used 15% reduction to achieve the accumulation of dislocations within the matrix of a WNiFe THA. Ravi Kiran et al. [125] applied cold RS with reductions of 10% and 30% and increased the UTS of a WNiFeCo THA without deteriorating its plasticity (with increasing swaging ratio, the UTS typically further increases, but the maximum elongation to failure decreases rapidly). 

**Figure 11:**
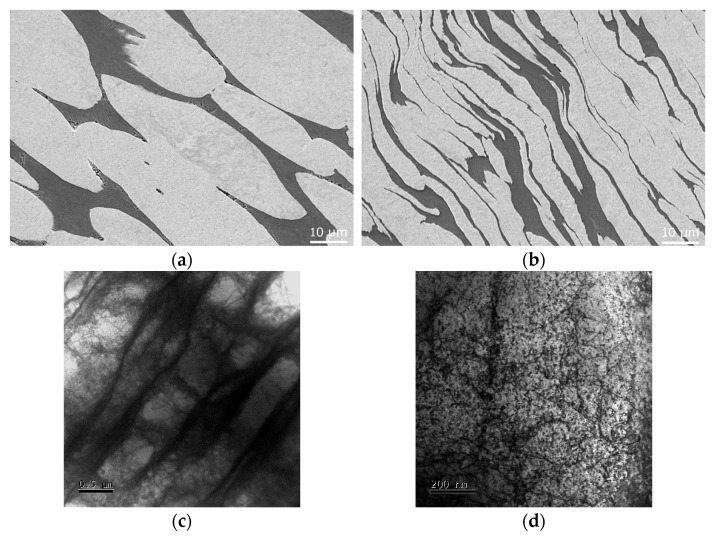
SEM-SE image of WNiCo microstructure, longitudinal cut: swaged at 900 °C, *φ* = 1.7 (**a**); swaged at 1 200 °C, *φ* = 1.7 (**b**). TEM bright field image of substructure within a longitudinal cut through W agglomerate: swaged at 20 °C, *φ* = 1.7 (**c**); swaged at 900 °C, *φ* = 1.7 (**d**) [original by author].

With continuing swaging, i.e., increasing the swaging ratio, W agglomerates start to gradually rotate in the direction of the main acting force, i.e., dominant plastic flow vector. Further increase in the swaging ratio then causes the agglomerates to elongate and gradually deform [126]; this effect can even be supported by decreasing the flow stress via elevating the processing temperature. Figure 11a depicts a SEM-SE image of a microstructure taken from a longitudinal cut through a WNiCo bar swaged at a temperature of 900 °C with a ratio of 1.7, while Figure 11b depicts a similar SEM-SE image of a WNiCo microstructure taken from a longitudinal cut through a bar swaged with an identical swaging ratio at 1 200 °C. The comparison of the SEM images clearly documents the positive effects of the elevated processing temperature on thedecreased flow stress of the NiCo matrix, and also the W agglomerates. Increasing the swaging ratio when swaging at elevated temperatures can even result in the generation of adiabatic shear bands (ASBs), as reported by, e.g., Sun et al. [127], who observed the formation of ASBs within a WNiFe THA subjected to a 40% reduction of RS performed at 700 °C. The processing of THAs with relatively higher swaging ratios causes the generation of a significant volume of dislocations and the formation of subgrains not only within the matrix but also within the W phase, as depicted in Figure 11c showing a TEM image of the dislocation substructure of a W agglomerate within a longitudinal cut through a WNiCo rod subjected to a swaging ratio of 1.7 at room temperature. As confirmed, elevating the swaging temperature enhances the formability of the W phase and thus enables the agglomerates to deform more easily. This fact is non-negligibly connected to the occurring structural phenomena, i.e., the annihilation and rearrangement of dislocations. This is documented in Figure 11d showing a TEM image of the dislocation substructure of a W agglomerate within a longitudinal cut through a WNiCo rod subjected to the swaging ratio of 1.7 at the temperature of 900 °C (same sample as in Figure 11a). 

The variables influencing the deformation behavior and performance of swaged THAs are numerous, e.g., exact chemical composition, deformation conditions, application of thermomechanical processing or heat treatment, etc. Moreover, as THAs feature two individual phases, their interaction will determine the final properties in each individual case. Nevertheless, the deformation behavior of THAs can be predicted based on empirical experience and experimental testing (e.g., [128]) but also based on simulations and modelling (e.g., [129]).

### 3.3. Metals with HCP Lattice

In accordance with the von Mises criterion, polycrystalline metallic materials require a minimum of five independent active slip systems for uniform plastic deformation to proceed via the dislocation slip mechanism [130]. However, metals featuring the HCP lattice only have three available slip systems, which limits their plasticity at room temperature. Therefore, to increase the formability of such metals, the <c+a> pyramidal slip system or twinning, both of which are sensitive to grain size, should be activated. Nevertheless, the type of the activated deformation mechanism is also directly related to the lattice parameters. 

For example, for Ti, the tendency to deform via the twinning mechanism gradually transforms into the tendency to perform the <c> slip with decreasing grain size [131]. Wang et al. [132] deformed grade 2 Ti via RS at room temperature with a swaging ratio of 2.77 and achieved a UFG microstructure with an average grain size of about 1 μm, which provided the Ti with exceptional strength and fatigue properties, but with very low plasticity. Nevertheless, by applying a very short annealing (5 min) at 450 °C, they imparted the annihilation of a substantial portion of dislocations and the formation of nano-sized subgrains (200–400 nm), which enhanced the plasticity up to the maximum elongation of 8.5% without losing the achieved strength. Molina-Aldareguia et al. [133] and Sabirov et al. [134] applied ECAP + RS + drawing to introduce nano-sized microstructures (grains of about 200 nm) within CP Ti, the UTS of which finally reached 1 280 MPa. Chuvil’deev et al. [135] introduced UFG microstructure with nano-sized precipitates within a TiAlZr alloy (UTS of 1 080 MPa) using room temperature RS with a swaging ratio of 2.4, while Dyakonov et al. [136] used RS, with a swaging ratio of 1.56, to process the VT8M-1 Ti-based alloy to achieve the homogeneous distribution of strengthening globular precipitates ~300 nm in size.

**Figure 12:**
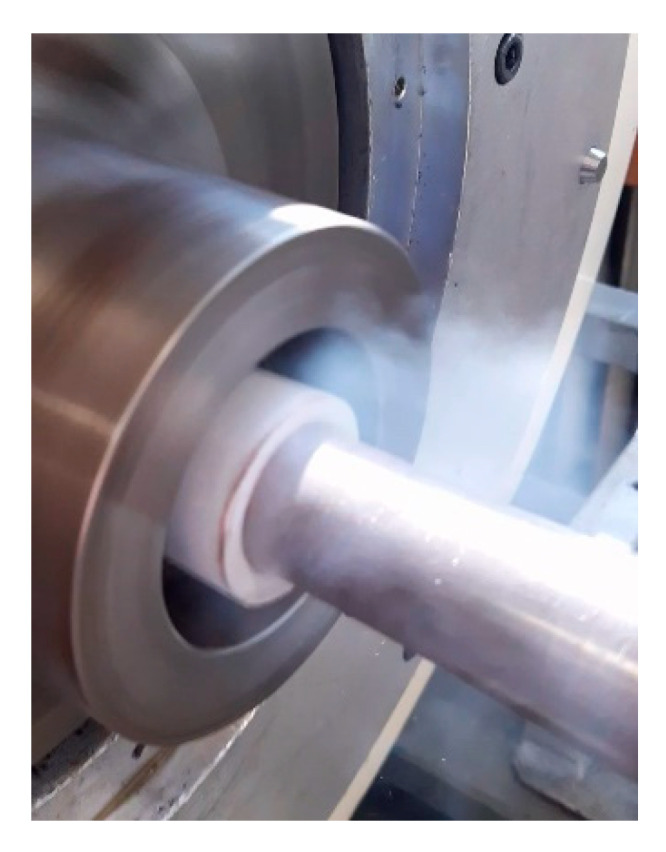
Rotary swaging of a Mg alloy workpiece at cryogenic conditions [original by author].

A similar effect of decreasing the grain size on the activation of the <c+a> slip was observed for Mg [137]. The plasticity of Mg is generally lower than that of Ti [138]. Nevertheless, given its favorable stress state, RS has successfully been applied to Mg (alloys) too. The importance of the activation of non-basal slip systems within Mg alloys during RS processing was emphasized by, e.g., Li et al. [139], who used room temperature RS to deform a MgAlCaMnZn alloy and achieve a strength of almost 400 MPa, as RS contributed significantly to the refinement of Al_8_Mn_5_ second phase precipitates. When subjected to deformation, Mg primarily exhibits twinning and the generation of stacking faults (all together with the generation of dislocations and the activation of slip systems), which gradually results in grain refinement; Yang et al. [140] performed a detailed study of the interactions of structural phenomena (i.e., twinning, stacking faults, and dislocations) within a MgLi alloy; in order to thoroughly map the occurring structural features, they gradually applied room temperature RS with a total swaging ratio of up to 0.32 in very low increments (swaging ratio of ~0.02 in a single pass). Comparable developments of structure-forming phenomena were also observed by Zhou et al. [141] for another MgLi alloy. A MgGdYZr alloy was studied by Wan et al. [142], who achieved a gradient microstructure featuring a final grain size of approx. 80 nm through a combination of RS at room temperature (35% total cross-section reduction), and subsequent ageing at 338 K for 72 h. For all the mentioned Mg-based materials, the highly deformed peripheral region of the workpiece typically featured a very high density of twins. Nanocrystallization induced by room temperature RS within MgY and AZ31 alloys was studied by Chen et al. [143], who concluded that the latter exhibited a higher grain refinement rate due to the higher activity of twining within its microstructure. In order to further optimize the microstructures vs. mechanical performance ratio of Mg alloys, RS can be applied at elevated/decreased temperatures. Wang et al. [144] used hot RS to optimize the development of dynamic recrystallization and demonstrate the formation of equiaxed grains in order to increase the ductility of a WE43 alloy. Hot RS was also applied to create (biocompatible) composites featuring combinations of Mg and Ti [145]. On the other hand, Chen et al. [146] used cryogenic RS to demonstrate a significant accumulation of twins and lattice defects within an AZ31B alloy to achieve exceptional strength. An example of a Mg-based workpiece being swaged at cryogenic conditions can be seen in Figure 12.

As for other HCP metallic materials, ZnMg and ZnMgCa biocompatible alloys subjected to RS at 200 °C were studied by Martynenko et al. [147]; they concluded that RS processing enhanced the UTS (~250 MPa) and corrosion resistance of the alloys but did not impair their biocompatibility. Alloys based on Zr were studied by Rogachev et al. [148], who subjected a ZrNb alloy (E125) to RS with a total reduction of 19% and subsequent annealing at 580 °C and achieved a UFG microstructure with homogeneous distribution of the β Nb phase. Ashida et al. [149], who studied the magnetic performance of a ZrMo alloy subjected to RS with up to 84% reduction, reported that, at high swaging ratios, both the fine-grained α and β phases were reoriented to the <10-10> direction, which contributed to substantial hardening while keeping favorable magnetic susceptibility. 

## 4. Conclusions and Outlook

As documented by numerous experimental works, RS has many advantages with regards to microstructure development and, consequently, also the mechanical and utility properties of swaged pieces. Contrary to SPD methods, which are advantageous when introducing highly refined microstructures but are typically limited to finite material volumes of workpieces, RS enables the processing of materials to improve their properties before additional processing, but it is also suitable for producing final components with enhanced performance and no need for final machining. Therefore, it is not only a time-effective but also a cost-effective industrially applicable process.

Room temperature RS has been proven to introduce the (substantial) strengthening of materials by imparting the accumulation of high densities of dislocations. Nevertheless, swaging under cryogenic conditions has the potential to enhance the effectivity of the process from the viewpoint of the accumulation of structure defects even more. For this reason, studying cryogenic swaging and its effects on microstructures is among the potential ways for future research. Also, as RS under cryogenic temperatures is supposed to aggravate plastic flow of the material and, at the same time, support the generation of dislocations in regions featuring the highest imposed strain, it can also be highly suitable for producing materials featuring gradient structures. Nevertheless, such processing would inevitably introduce (large) stress gradients, and thus the optimization of the processing from the viewpoint of residual stress should also be within the focus of future research, as stress gradients and inhomogeneities can decrease the endurance and fatigue life of the swaged components.

## Data Availability

As this is a review paper, the original supporting data are publicly available, except the figures created by the author, which can be available on request from the author.
